# Egg product freshness evaluation: A metabolomic approach

**DOI:** 10.1002/jms.4256

**Published:** 2018-07-30

**Authors:** Daniele Cavanna, Dante Catellani, Chiara Dall'Asta, Michele Suman

**Affiliations:** ^1^ Advanced Laboratory Research Barilla G. e R. Fratelli S.p.A. Parma Italy; ^2^ Department of Food and Drug University of Parma Parma Italy

**Keywords:** egg product, freshness, LC‐HRMS, metabolomics, nontargeted mass spectrometry

## Abstract

Egg products' freshness is a crucial issue for the production of safe and high‐quality commodities. Up to now, this parameter is assessed with the quantification of few compounds, but the possibility to evaluate more molecules simultaneously could help to provide robust results.

In this study, 31 compounds responsible of freshness and not freshness of egg products were selected with a metabolomic approach. After an ultrahigh‐pressure liquid chromatography–high‐resolution mass spectrometry (UHPLC‐HRMS) analysis, different chemometric models were created to select gradually the most significant features that were finally extracted and identified through HRMS data.

Sample lots were collected directly from their arrival at the production plant sites, extracted immediately after, then left at room temperature, and extracted again after 24 and 48 hours (first day and second day, respectively). A total amount of 79 samples was used for the model creation.

Furthermore, the same compounds were detected in seven new egg products sample lots not used for the model creation and treated with the same experimental design (total amount of samples, 21).

The results obtained clearly demonstrate that these 31 molecules can be considered real freshness or not freshness chemical markers.

Furthermore, this UHPLC‐HRMS metabolomic approach allows for the detection of a larger set of metabolites clearly related to possible microbial growth over time, which is a relevant point for also ensuring food safety.

## 1 INTRODUCTION

Starting from the beginning of the new millennium, consumers' attention on the authenticity and quality of food commodities has strongly increased, leading to an increase in the demand of fit‐for‐purpose methods for detecting food fraud from both industries and research institutes.

Eggs, mostly in the egg products form, are largely used for the formulation of food products. Freshness is therefore a crucial parameter for ensuring the production of safe and high‐quality commodities. However, the assessment of egg freshness is still challenging, due to the lack of robust chemical markers.

In the past, according to the European legislation, two parameters typical of microbiological fermentation had to be monitored: lactic acid (≤1000 mg/kg of dry egg) and succinic acid (≤25 mg/kg of dry egg),^1^ by the current laws consider only lactic acid as reliable marker.^2^

These two compounds could act sometimes as “tardive” markers of eggs ageing, and their increase is often not linear during the time; moreover, the use of only one/two molecule(s) for the evaluation of such critical parameter can be not sufficient in some circumstances; at the same time, more “not freshness” compounds should be monitored.

Up to now, major attention was paid to the development of rapid techniques able to assess egg products freshness, eg, using electronic noses^3^, ^4^, ^5^ or spectroscopic analyses.^6^, ^7^, ^8^ These techniques are particularly suitable for industrial screening purposes, due to the reduced time and costs.

However, the identification of robust “markers of freshness” (ie, compounds that decrease their intensity during the egg products storage) could strongly support the evaluation of egg products ageing, with a reduction of the risk for the final consumer.

In this context, the use of “nontargeted” methods based on a metabolomic approach—which are considered emerging methodologies for detecting food frauds^9^—will offer the opportunity to identify and validate proper markers.

In consideration of the ageing process, chances in the volatile profile of egg products can be expected. Therefore, among possible analytical techniques, gas chromatography–mass spectrometry could be used for the identification of these changes, in agreement with previous applications in the field of food frauds, ie, tomato cultivars discrimination,^10^ honey authenticity,^11^ or geographical origin of saffron.^12^

However, the nonvolatile fraction of food may be of great interest for the discrimination of ageing.

Scientific papers suggest that other rapid mass spectrometry approaches, as, for example, direct analysis real‐time–mass spectrometry^13^ or rapid evaporative ionization–mass spectrometry,^14^ could lead to the characterization of raw materials, avoiding the chromatographic separation.

In any case, liquid chromatography–high‐resolution mass spectrometry (LC‐HRMS) is the technique of choice for the execution of a metabolomic study: Thanks to its versatility, a huge amount of information related to the nonvolatile profile can be extracted from the chromatographic fingerprints.

Literature presents applications of this approach for the detection of frauds related to a wide range of raw materials, from fruit juices^15^ to wheat^16^ and from extra virgin olive oil^17^ to spirit drinks.^18^

Independently from the analytical technique used, the creation of robust chemometric models is a crucial step for the selection of new marker compounds responsible of the target fraud.^19^

Based on what described above, this work presents a metabolomic study on egg products samples able to select and identify a group of new compounds that can be used as “freshness” or “not freshness” markers.

Liquid chromatography–high‐resolution mass spectrometry was exploited for fingerprints recording; subsequently, robust data elaboration was executed, creating different chemometric models able to select some marker compounds that were then studied and identified.

The final step of this workflow was the validation of these molecules, with the aim to assess their value as chemical markers, independently from the chemometric model used to select them.

## 2 MATERIALS AND METHODS

### 2.1 Chemicals and sample description

Methanol, acetonitrile, formic acid (FA), and ammonium formate (AF) were purchased from VWR International, Ltd (Poole, UK).

Analytical standards of chloramphenicol, lactic acid, succinic acid, phenyllactic acid, tyramine, uracil, asparagine, glutamine, guanosine, guanosine monophosphate, serine, uridine, and uridine monophosphate were purchased from Sigma‐Aldrich (St Louis, Missouri).

Water was purified using a Milli‐Q system (Millipore, Bedford, Massachusetts).

Egg products samples were directly collected from batches that daily arrive at production plant sites; to increase the variability of the model, the sampling period lasted 6 weeks, and samples coming from different batches, different suppliers, and with different amounts of carotenoids (in the range of tenths of milligrams per kilogram, authorized for improving appearance and correspondent consumer preference in finished products) were selected.

### 2.2 Experimental design

The first step of the adopted workflow, as suggested by US Pharmacopoeia,^20^ required the creation of chemometric models able to discriminate between fresh and not fresh samples; subsequently, the features responsible of this clusterization were selected, and a tentative of compounds identification was performed.

Finally, marker compounds identified were validated, that means that the target molecules were searched in new egg products batches subject to the same ageing applied during the model creation.^15^

For the creation of the model, 29 egg products batches were collected and extracted immediately after the reception at the production plant site; subsequently, these raw materials were left at room temperature and extracted again after 1 day and after 2 days, according to the sampling plan described in Table 1. Summarizing, a global amount of 79 samples was used for the creation of the chemometric model.

**Table 1:** Sampling plan for the model creation

Sample	0 h	1 d	2 d
1	X^a^	X	X
2	X	X^a^	X
3	X	X	X^a^
4	X	X	X
5	X	X	X
6	X^a^	X	X
7	X	X	X
8	X	X	
9	X	X	
10	X	X^a^	X
11	X	X	X^a^
12	X	X	X
13	X	X	X^a^
14	X	X^a^	X
15	X	X	X^a^
16	X	X	
17	X^a^	X	
18	X	X	
19	X	X^a^	X
20	X	X	X
21	X	X	X^a^
22	X^a^	X	X
23	X	X^a^	X
24	X	X	X
25	X	X^a^	X
26	X^a^	X	X
27	X	X	
28	X	X^a^	
29	X^a^	X	

a Double sample prep.

For the validation of the marker compounds, seven new egg products batches were collected, extracted, and analyzed following the same experimental design described for the model creation: The total amount of samples was 21.

### 2.3 Sample preparation

A total of 300 μL of each egg product at each time point was spiked into a 1.5‐mL tube, together with 30 μL of a 20‐μg/mL chloramphenicol solution (used as internal standard) and 900 μL of a mixture of acetonitrile to water in the ratio 80:20 (stored at 2°C‐8°C).

This mixture was vortexed for 1 minute, then stored for 5 minutes at −20°C, and subsequently centrifuged for 10 minutes at 14 000 rpm with a Rotina 380R (Hettic Lab Technology, Tuttlingen, Germany) maintained at 4°C.

The extract was filtered into a high‐performance liquid chromatography vial with a 0.22‐μm PolyTetraFluoroEthylene (PTFE) syringe filter (Phenomenex, Torrance, California) and stored at −20°C until analysis with LC‐HRMS instrument.

For the evaluation of method reproducibility, 20% of the samples were double prepared, as detailed in Table 1.

During each extraction session, the same procedure was performed also into empty tubes, in which all the steps were executed without the egg product addition. These samples were labelled as “extraction blanks.”

A 500‐ng/mL chloramphenicol solution in acetonitrile to water ratio of 80:20 was prepared and named “standard solution.”

Additionally, two quality control (QC) samples were prepared mixing 10 μL of each extract sample.

### 2.4 LC‐HRMS analysis

High‐performance liquid chromatography analysis was performed with a Dionex UltiMate 3000 ultrahigh‐performance liquid chromatography (Thermo Fisher Scientific, Inc, Waltham, Massachusetts) equipped with a Luna Omega 150 mm × 2.1 mm, 1.6 μm particle size analytical column (Phenomenex, Torrance, California) with a precolumn security guard ultra C18, both maintained at 30°C. Gradient elution was performed using FA and AF as mobile phase modifiers with a constant flow rate of 0.3 mL/min.

Gradient conditions are the following: After 1 minute with 95% of mobile phase A (0.1% FA and 5mM AF in water) and 5% of mobile phase B (0.1% FA and 5mM AF in methanol), the percentage of solvent B increased to 95% in 24 minutes and then was maintained at this percentage for 5 minutes before column re‐equilibration (10 min).

Autosampler was maintained at 5°C, and the injection volume was 5 μL.

Mass spectrometry detection was performed with a benchtop Q Exactive Hybrid Quadrupole‐Orbitrap mass spectrometer (Thermo Fisher Scientific, Waltham, Massachusetts) equipped with a heated (H) electrospray ionization (ESI) interface (Thermo Fisher Scientific, Waltham, Massachusetts). Two analytical sequences (one with positive and one with negative ionization mode) were executed with a “Full Scan‐data dependent fragmentation” experiment.

Source conditions were as follows: sheath and auxiliary gas flow rates of 40 and 20 arbitrary units, respectively; heater temperature of 250°C with a spray voltage of 3.2 kV (ESI pos) and −3.0 kV (ESI neg). The capillary was kept at 220°C and the S‐lens RF level was set at 55 AU for both the acquisition modes.

The full‐scan accurate mass spectra from 75 to 1000 Da for both the ionization modes were obtained with a resolution of 70 000 Full Width at Half Maximum (FWHM) (*m/z* 200), automatic gain control target 1e6, and maximum injection time of 200 milliseconds.

In all the experiments, the data‐dependent tandem mass spectrometry (MS/MS) acquisition was executed with a resolution of 17 500 FWHM (*m/z* 200) and intensity threshold 6e4. The quadrupole isolation window was kept at 2.0 *m*/*z* with a TopN value of 5. The scan range was from 50 to the fragmented mass *m*/*z* (*m*/*z* +25), automatic gain control target 2e5, maximum injection time of 50 milliseconds, and normalized collision energy of 30% with ±50% step.

Samples, together with the “extraction blanks,” were randomly injected to avoid systematic bias.^16^ The “standard solutions” and the QC samples were injected at the beginning of the sequences and every 10 sample injections.

### 2.5 Data treatments and statistics

Ultrahigh‐performance liquid chromatography–high‐resolution mass spectrometry raw data were acquired using Xcalibur software (version 3.0, Thermo Fisher Scientific, Waltham, Massachusetts); peaks alignment, “extraction blanks” subtraction, and features extraction were performed using Compound Discoverer software (version 2.0 Thermo Fisher Scientific, Waltham, Massachusetts); the mass range inspected was between 75 *m/z* and 1000 *m/z* from 1 to 30 minutes of the chromatographic runs.

The values of the critical parameters for features extractions are the following: precursor ion deviation of 5 ppm (for the positive runs) and 10 ppm (for the negative runs); maximum retention time shift of 0.3 minutes; minimum peak intensity for a peak to be selected 1 000 000 AU; relative intensity tolerance used for isotope search 30%.

Structures prediction was also performed using “ChemSpider” databases, setting a maximum mass shift of 5 ppm for positive acquisitions and 10 ppm for negative acquisitions.

For the “*m/z CLOUD*” MS/MS library search, the precursor mass tolerance used was 0.05 Da while the fragments mass tolerance was 10 ppm.

The resulting two data matrixes (for positive and negative ionization modes), containing the area values provided by Compound Discoverer for all the features, were exported and processed with SIMCA software (version 14.1 Umetrics, Umea, Sweden) for chemometric data elaboration.

Data were log transformed and Pareto scaled, and then a preliminary principal component analysis (PCA) was executed to check the clusterization of the samples and the QCs positioning in the scores plot.

Subsequently, features were filtered, and only the ones that had a Coefficient of Variation (CV)% lower than 40% in the QC samples were selected.

A new PCA was executed to check the expected improvement in samples separation and QCs positioning; after the evaluation of the replicate samples placement in the scores plot, a final PCA was calculated without the QC samples and with the average values of the replicates.

Afterwards, supervised orthogonal partial least square discriminant analysis (OPLS‐DA) models were built comparing the “fresh” samples against the “1 day” samples, against the “2 days” samples and against the union of “1 day” and “2 days” samples.

Thanks to the S‐plots, statistically significant markers responsible of the clusterization were selected: The ions furthest away from the origin contribute more significantly to the separation between the groups and may therefore be regarded as the differentiating ones.^21^ This approach was used for the six OPLS‐DA models to be sure that all the most discriminative features could be selected.

In addition, their VIP (variable influence on projection) values were evaluated to assess their relevance for the chemometric model (VIP values had to be >1.4).^22^

Features that survived this process were studied, and a tentative of compounds identification was performed.

In all the chemometric models created, an internal leave 1/7 out cross‐validation was executed.

### 2.6 Compounds identification workflow

Compounds identification was performed according to the following steps:

1. Chemical formula hypothesis, taking into account also adducts, if present.
2. Retention time evaluation.
3. Evaluation of the mass shift between the experimental and the theoretical values.
4. Isotopic pattern evaluation and hypothesis of a chemical structure referring also to ChemSpider databases.
5. MS/MS spectra study and comparison between the theoretical fragmentations (performed with Compound Discoverer) and the fragments detected.
6. MS/MS spectra comparison with the *m*/*z* CLOUD database (in which fragmentations with other Orbitrap instruments are available).
7. If available, Reference Standard injection as final confirmation.

The Standard Initiative in Metabolomics^23^ and subsequent improvements^24^ suggest different ranks of compounds identification according to the number of steps completed: identification steps completed: 1 to 5, level of identification: 3; identification steps completed: 1 to 6, level of identification: 2; identification steps completed: 1 to 7, level of identification: 1; this classification was used for the selected features.

### 2.7 Markers validation procedure

The compounds identified with the workflow detailed above were searched in seven new egg products batches not used for the model creation; they were collected, extracted, and analyzed with the same experimental design previously described.

The maximum reliability of these compounds as “freshness” or “not freshness” markers implies they have to be confirmed not only with their presence or absence in the new samples but also highlighting the same increasing or decreasing trend through the time points.

## 3 RESULTS AND DISCUSSION

### 3.1 Extraction procedures and sequences evaluation

Before starting the real data elaboration, different evaluations on the internal standard results were performed.

Chloramphenicol is an exogenous compound that was added to every sample with the aim of monitoring the extraction procedures and the goodness of the analytical sequences.^25^

The injection of the “standard solution” periodically during the sequence provided information about the sequence trends and helped in the detection of potential decrease in signal's intensity.

For this reason, at the end of each analytical sequence, the CV% of both areas and retention times of chloramphenicol peak in the “standard solution” injections (11 for each sequence) were calculated. For the sequence analyzed in positive ionization mode, the values obtained were 10.8% (areas) and 2.2% (retention time) and, for the negative ionization mode, 10.1% (areas) and 2.8% (retention time).

The results obtained highlight that the response of the instrument was acceptable during the whole sequences (126 injections each) for both positive and negative ionization modes.

An analogous evaluation of chloramphenicol peaks intensity and retention times shift was performed for all the sample injections: In this way, more information about the goodness of the analytical sequences were obtained; moreover, the CV% of the area values of the internal standard was used for the extraction reproducibility evaluation, assuring that no mistakes occurred during samples preparation.

For the sequence analyzed in positive ionization mode, the areas CV was 7.2%, and the retention time CV was 1.6% and, for the negative ionization mode, 10.5% (areas) and 3.1% (retention time).

According to the results obtained, we can deduce that no issues occurred during sample extraction procedures; moreover, the slight shift in the retention times is a further proof of the goodness of the analytical sequences.

### 3.2 Chemometric data elaboration

The preliminary PCA obtained with the areas of the entire data set highlighted a separation between the fresh samples and the not fresh ones with a clustering of the QC samples for both positive and negative ionization modes (data not shown); the first two components described more than 45% of the variance for both the models.

**Figure 1:**
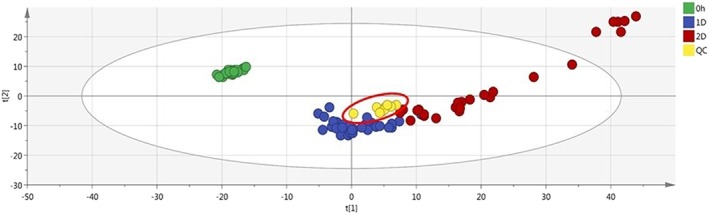
ESI + PCA scores plot of the samples (n = 107) after features filtering. Leftmost area dots (0 h), fresh samples; 1D dots, “1 day” samples; 2D dots, “2 days” samples; Quality Control (QC) dots, “QC samples” (circled). Explained variance of the first two PCs, 55.6%. ESI, electrospray ionization; PCA, principal component analysis; QC, quality control

**Figure 2:**
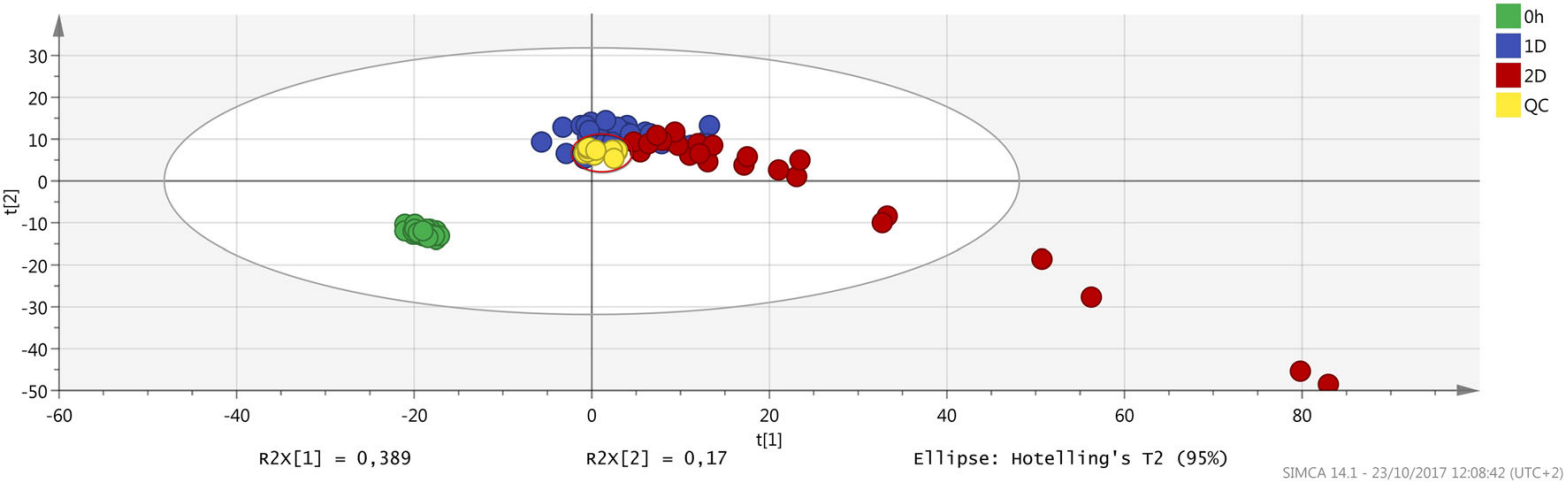
ESI − PCA scores plot of the samples (n = 107) after features filtering. Leftmost area dots (0 h), fresh samples; 1D dots, “1 day” samples; 2D dots, “2 days” samples; QC dots, “QC samples” (circled). Explained variance of the first two PCs, 55.9%. ESI, electrospray ionization; PCA, principal component analysis; QC, quality control

After data filtering (obtained by selecting in the QC samples the features with CV lower than 40% in the area values), an improvement in the clusterization of the samples was obtained, with a tight clustering of the QC samples (Figures 1 and 2). In addition, the overlay in the scores plot of the replicate extracted samples was essentially complete.

The tight clustering of the QC samples in the center of the score plot is a further confirmation of the robustness of the analytical procedure; moreover, this ensure that the separation through the groups is not random but due to a real variability.^26^

The overlay in the scores plot of the replicate samples certify that the extraction procedure is repeatable not only for the chloramphenicol compound but for the whole fingerprint of the egg product.

**Figure 3:**
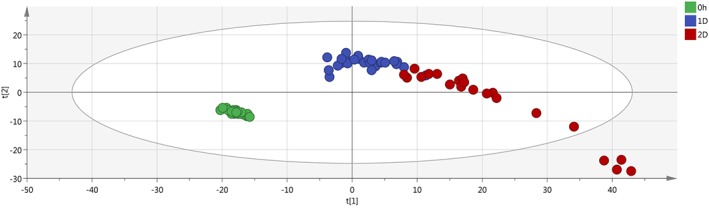
Final ESI + PCA scores plot of the samples. Leftmost area dots (0 h), fresh samples; 1D dots, “1 day” samples; 2D dots, “2 days” samples. Explained variance of the first two PCs, 57.3%. ESI, electrospray ionization; PCA, principal component analysis; QC, quality control

**Figure 4:**
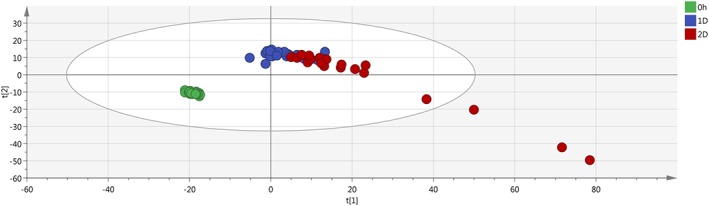
Final ESI − PCA scores plot of the samples. Leftmost area dots (0 h), fresh samples; 1D dots, “1 day” samples; 2D dots, “2 days” samples. Explained variance of the first two PCs, 58.2%. ESI, electrospray ionization; PCA, principal component analysis; QC, quality control [Colour figure can be viewed at wileyonlinelibrary.com]

The final PCA, created with the average value of the replicates and without the QC samples, clearly highlights the separation between the fresh samples and the not fresh ones (Figures 3 and 4).

**Figure 5:**
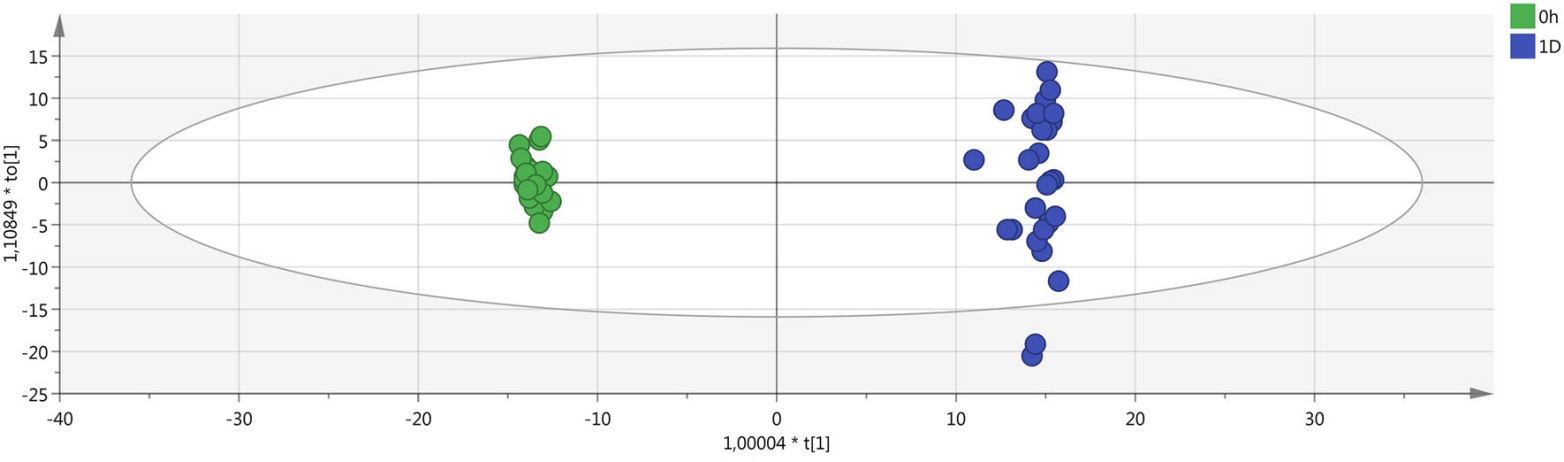
ESI + OPLS‐DA scores plot of the fresh samples against the “1 day” samples. Left area dots (0 h), fresh samples; right area dots (1D), “1 day” samples. ESI, electrospray ionization; OPLS‐DA, orthogonal partial least square discriminant analysis [Colour figure can be viewed at wileyonlinelibrary.com]

**Figure 6:**
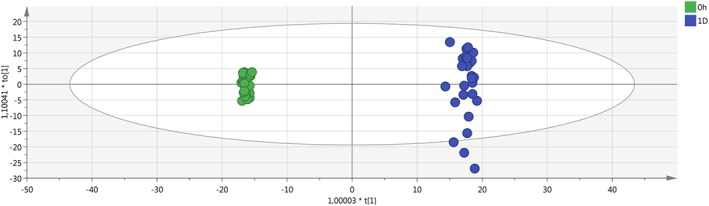
ESI − OPLS‐DA scores plot of the fresh samples against the “1 day” samples. Left area dots (0 h), fresh samples; right area dots (1D), “1 day” samples. ESI, electrospray ionization; OPLS‐DA, orthogonal partial least square discriminant analysis [Colour figure can be viewed at wileyonlinelibrary.com]

All the OPLS‐DA–supervised models, as expected, extraordinarily increase the separation between the two groups. As example, Figures 5 and 6 present the OPLS‐DA models created between the fresh samples and the “1 day” samples.

Tables 2 and 3 summarize the variance of the x and y variables explained by the models previously described—R^2^X (cum) and R^2^Y (cum)—and the percent of variation of the training set predicted by the model (Q^2^) according to cross‐validation.

**Table 2:** Global value of R^2^X (cum), R^2^Y (cum), and Q^2^ parameters for positive ionization mode

Model	PCA (Preliminary)	PCA (Filtered)	PCA (Final)	OPLS‐DA (“Fresh” vs “1D + 2D”)	OPLS‐DA (“Fresh” vs “1D”)	OPLS‐DA (“Fresh” vs “2D”)
R^2^X (cum)	0.691	0.750	0.725	0.618	0.589	0.680
R^2^Y (cum)	/	/	/	0.992	0.997	0.999
Q^2^	0.544	0.643	0.590	0.989	0.990	0.994

Abbreviations: OPLS‐DA, orthogonal partial least square discriminant analysis; PCA, principal component analysis.

/ means data available only in OPLS‐DA mode and not in PCA mode.

**Table 3:** Global value of R^2^X (cum), R^2^Y (cum), and Q^2^ parameters for negative ionization mode

Model	PCA (Preliminary)	PCA (Filtered)	PCA (Final)	OPLS‐DA (“Fresh” vs “1D + 2D”)	OPLS‐DA (“Fresh” vs “1D”)	OPLS‐DA (“Fresh” vs “2D”)
R^2^X (cum)	0.750	0.769	0.777	0.617	0.627	0.698
R^2^Y (cum)	/	/	/	0.994	0.997	0.999
Q^2^	0.584	0.577	0.546	0.991	0.991	0.996

Abbreviations: OPLS‐DA, orthogonal partial least square discriminant analysis; PCA, principal component analysis.

The results obtained strongly indicate that the models created for both positive and negative ionization modes are reliable and robust.

### 3.3 Compounds identification

Features responsible of the clusterization were selected, and a group of them was identified according to what described in the “compounds identification workflow” paragraph.

Both markers of “not freshness” (that drastically increase their intensity or even appear during the eggs ageing) and of “freshness” (that drastically decrease their intensity or even disappear during the eggs ageing) were identified.

Tables 4 and 5 summarize the compounds responsible of the “freshness” and “not freshness” classification, ranked according to the level of identification suggested by the Standard Initiative in Metabolomics.^23^, ^24^

**Table 4:** Resume of “not freshness” markers identified

Name	Pseudomolecular Ion	Detected *m*/*z*	RT, min	Predicted Formula	Mass Error, ppm	CV IN QCs, %	VIP Value	ID Type
Lactic acid	[M − H]^−^	89.0244	1.73	C3 H6 O3	12.4^d^	27	2.59^c^	1
Phenyllactic acid	[M − H]^−^	165.0555	11.95	C9 H10 O3	5.45	11	2.64^b^	1
Succinic acid	[M − H]^−^	117.0193	3.22	C4 H6 O4	8.55	11	2.49^b^	1
Tyramine	[M + H]^+^	138.0913	3.65	C8H11NO	0.72	30	2.21^b^	1
	[M + H–NH3]^+^	121.0650			1.65			
Uracil	[M − H]^−^	111.0196	1.70	C4 H4 N2 O2	6.31	15	2.88^c^	1
4‐Hydroxybutyric acid	[M − H]^−^	103.0398	3.90	C4 H8 O3	8.74	23	1.83^b^	2
6‐Methylquinoline	[M + H]^+^	144.0807	8.39	C10 H9 N	0.69	7	2.27^b^	2
d‐Alanyl‐d‐alanine	[M − H]^−^	159.0773	1.34	C6 H12 N2 O3	5.66	12	1.88^b^	2
Hydroxycaproic acid	[M − H]^−^	131.0712	11.25	C6 H12 O3	7.63	17	3.57^c^	2
Phosphorylethanolamine	[M − H]^−^	140.0118	1.14	C2 H8 N O4 P	7.85	18	3.00^c^	2
3‐Thiomorpholinecarboxylic acid	[M + H]^+^	148.0426	1.70	C5 H9 N O2 S	0.68	29	2.09^a^	3
Arginine‐proline dipeptide	[M + H]^+^	272.1716	1.30	C11 H21 N5 O3	0.37	13	2.55^c^	3
Lysine leucine dipeptide	[M − H]^−^	258.1818	1.70	C12 H25 N3 O3	2.32	16	1.81^b^	3
*N*‐Acetylhistidine	[M − H]^−^	196.0725	1.43	C8 H11 N3 O3	4.08	17	2.20^b^	3
2‐Hydroxyisovaleric acid or 2‐hydroxymethylbutyric acid	[M − H]^−^	117.0557	7.15	C5 H10 O3	9.39	18	2.15^c^	3
Mannose 6‐phosphate or glucose 6‐phosphate	[M − H]^−^	259.0226	1.08	C6 H13 O9 P	5.01	32	2.99 c	3

Abbreviations: OPLS‐DA, orthogonal partial least square discriminant analysis; QC, quality control; RT, retention time; VIP, variable influence on projection.

a VIP value obtained from the OPLS‐DA model 0 h vs all.

b VIP value obtained from the OPLS‐DA model 0 h vs 2D.

c VIP value obtained from the OPLS‐DA model 0 h vs 1D.

d This value is higher than the limit (10 ppm) but is due to the low molecular weight of lactic acid. The injection of the reference standard certified its identity beyond any reasonable doubt.

**Table 5:** Resume of “freshness” markers identified

Name	Pseudomolecular Ion	Detected *m*/*z*	RT, min	Predicted Formula	Mass Error, ppm	CV in QCs, %	VIP Value	ID Type
Asparagine	[M + H]^+^	133.0608	1.21	C4 H8 N2 O3	0.30	14	2.35^b^	1
Glutamine	[M + H]^+^	147.0762	1.23	C5 H10 N2 O3	1.36	8	2.75^b^	1
	[M − H]^−^	145.0615	1.17		4.83	12	2.55^b^	
Guanosine	[M − H]^−^	282.0844	4.85	C10 H13 N5 O5	3.90	33	2.88^b^	1
Guanosine monophosphate (GMP)	[M − H]^−^	362.0506	1.66	C8 H19 N3 O9 P2	1.93	28	3.10^b^	1
Serine	[M + H]^+^	106.0502	1.23	C3 H7 N O3	2.83	8	2.67^b^	1
Uridine	[M − H]^−^	243.0619	1.68	C9 H12 N2 O6	2.88	16	2.85^b^	1
Uridine monophosphate	[M − H]^−^	323.0285	1.37	C9 H13 N2 O9 P	3.10	17	3.20^b^	1
Arginine	[M + H]^+^	175.1189	1.22	C6 H14 N4 O2	0.22	16	2.15^a^	2
	[M − H]^−^	173.1038	1.17		2.89	9	2.38^a^	
Aspartic acid	[M + H]^+^	134.0448	1.22	C4 H7 N O4	0.03	10	1.72^b^	2
Histidine	[M + H]^+^	156.0768	1.20	C6 H9 N3 O2	0.02	20	1.69^b^	2
Methionine sulfoxide	[M + H]^+^	166.0530	1.28	C5 H11 N O3 S	1.20	12	1.68^b^	2
Methylhistidine	[M + H]^+^	170.0924	1.22	C7 H11 N3 O2	0.06	37	2.50^b^	2
Threonine	[M + H]^+^	120.0656	1.25	C4 H9 N O3	0.80	8	1.42^a^	2
Guanosine 5′‐diphospho‐d‐mannose or Guanosine 5′‐diphospho‐d‐glucose	[M − H]^−^	604.0694	1.30	C29 H17 N7 O5 P2	1.99	27	2.77^b^	3
*N*‐Acetyl‐α‐d‐galactosamine or *N*‐acetyl‐α‐d‐glucosamine	[M + H]^+^	222.0973	1.38	C8 H15 N O6	0.45	17	1.87^b^	3

Abbreviations: OPLS‐DA, orthogonal partial least square discriminant analysis; QC, quality control; RT, retention time; VIP, variable influence on projection.

VIP value obtained from the OPLS‐DA model 0 h vs all.

a VIP value obtained from the OPLS‐DA model 0 h vs 2D.

b VIP value obtained from the OPLS‐DA model 0 h vs 1D.

Twelve compounds were identified with their respective reference standards, and a total amount of 31 markers (15 of freshness and 16 of not freshness) was selected.

Table 6 resumes the number of features filtered out through each statistical step.

**Table 6:** Resume of the features selected after each statistical step

Data Analysis Step	Number of Features Selected	
	Positive Ionization Mode	Negative Ionization Mode
Peak alignment	3452	4893
Filtration according to CV% in QC	2189	3407
Extracted features from S‐plots	207	240
Final list	14	19

Abbreviation: QC, quality control.

As highlighted in the markers lists, lactic acid and succinic acid were undoubtedly detected, but together with many other compounds. This could lead to a more complete evaluation of the freshness issue: potentially 31 *m*/*z* values can be simultaneously considered for this topic.

### 3.4 Markers validation

As also suggested by the US Pharmacopoeia,^20^ predictive chemometric models should be always validated with an external set of samples (possibly collected in different periods) that have to be treated as unknown; their classification should be predicted, to certify that the samples clustering is real and not related to some overfitting of the chemometric model.

This approach is hard to perform with metabolomic results: Because of the ultrahigh‐performance liquid chromatography–high‐resolution mass spectrometry intrinsic variability, only chromatograms acquired with the same analytical sequence can be aligned and studied; the use of chemometric model to predict the classification of samples acquired with different sequences could lead to dangerous mistakes. Moreover, the goal of this work was the identification of new markers of freshness, and the chemometric model was considered only a tool to reach this goal.

For these reasons, the marker compounds and not the model were validated, that means that all these 31 features were searched in the new samples and their presence or absence, together with their trends through the time points, were evaluated and compared with the results obtained during the model creation, as presented in Tables 7 and 8.

**Table 7:** Comparison of the mean area values (+/− standard error) of not freshness markers between the model creation and the markers validation^a^

Compound Name	Model Creation	Marker Validation
Lactic acid	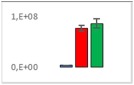	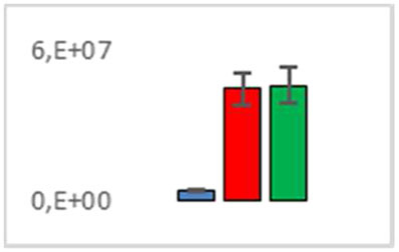
Phenyllactic acid	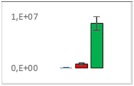	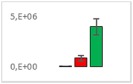
Succinic acid	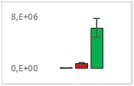	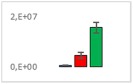
Tyramine	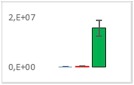	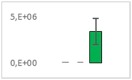
Uracil	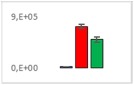	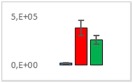
2‐Hydroxyisovaleric acid or 2‐hydroxymethylbutyric acid	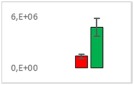	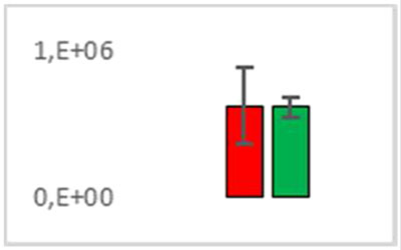
4‐Hydroxybutyric acid	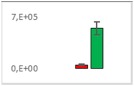	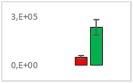
6‐Methylquinoline	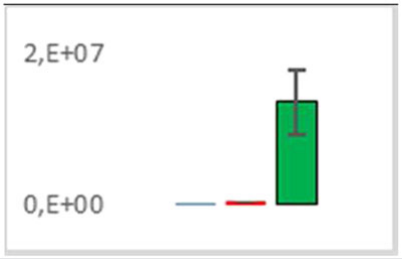	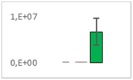
d‐Alanyl‐d‐alanine	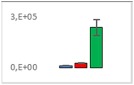	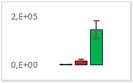
Hydroxycaproic acid	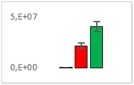	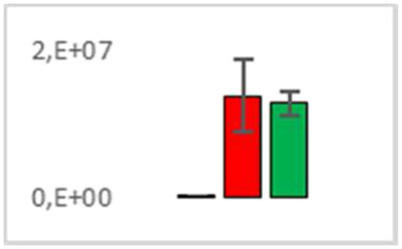
Mannose 6‐phosphate or glucose 6‐phosphate	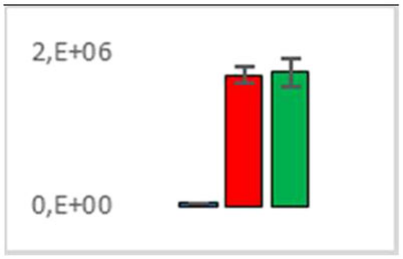	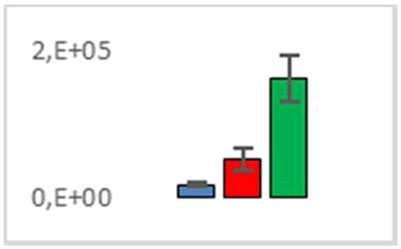
Phosphorylethanolamine	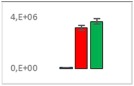	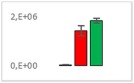
3‐Thiomorpholinecarboxylic acid	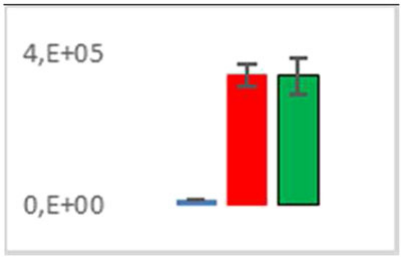	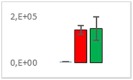
Arginine‐proline dipeptide	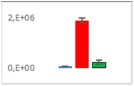	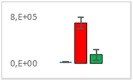
Lysine leucine dipeptide	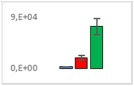	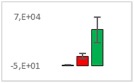
*N*‐Acetylhistidine	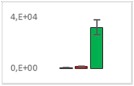	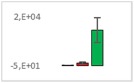

a Time points: first bar, “*t* zero”; second bar, “1 day”; third bar, “2 days.”

**Table 8:** Comparison of the mean area values (+/− standard error) of freshness markers between the model creation and the markers validation^a^

Compound Name	Model Creation	Marker Validation
Asparagine	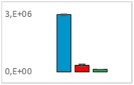	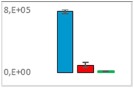
Glutamine (ESI+)	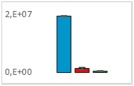	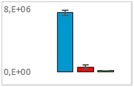
Glutamine (ESI−)	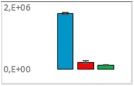	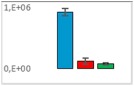
Guanosine	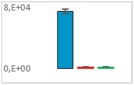	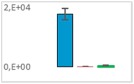
Guanosine monophosphate	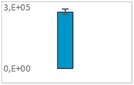	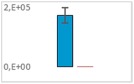
Serine	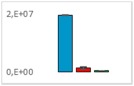	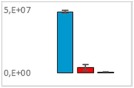
Uridine	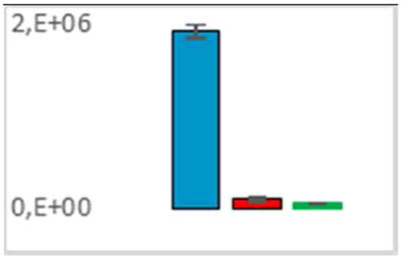	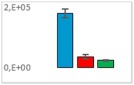
Uridine monophosphate	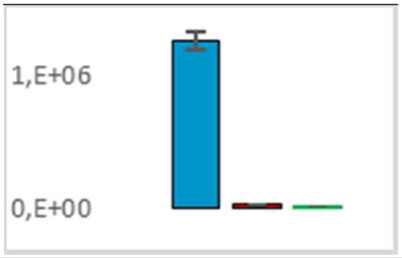	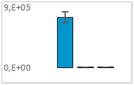
Arginine (ESI+)	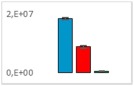	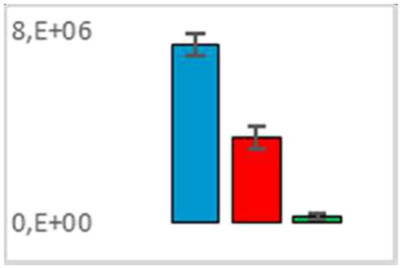
Arginine (ESI−)	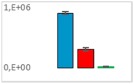	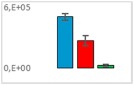
Aspartic acid	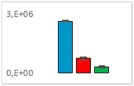	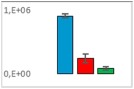
Guanosine 5′‐diphospho‐d‐mannose or guanosine 5′‐diphospho‐d‐glucose	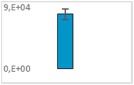	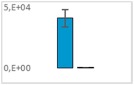
Histidine	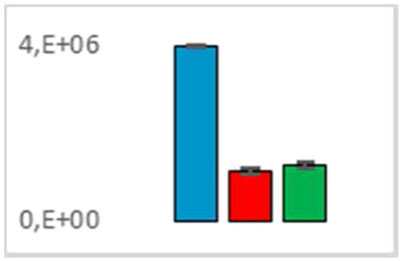	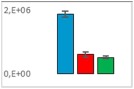
Methionine sulfoxide	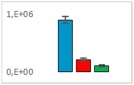	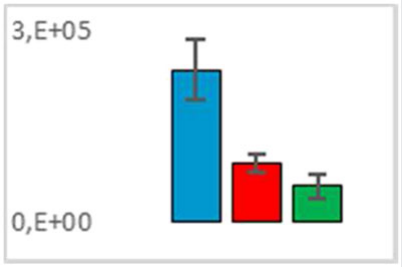
Methylhistidine	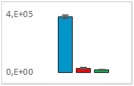	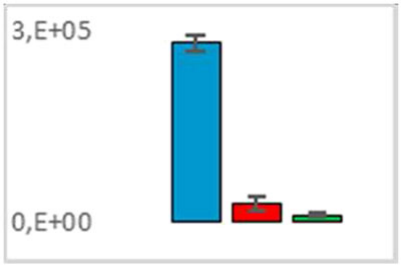
*N*‐Acetyl‐α‐d‐galactosamine or *N*‐acetyl‐α‐d‐glucosamine	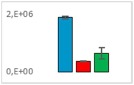	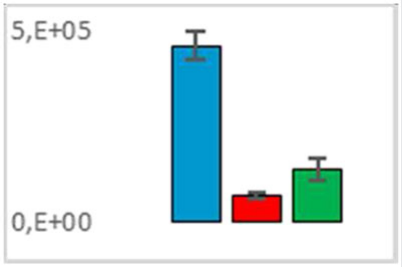
Threonine	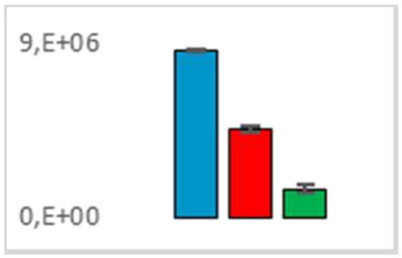	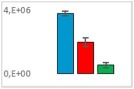

a Time points: first bar, “*t* zero”; second bar, “1 day”; third bar, “2 days.”

All the target molecules were detected with almost the same trend through the time points (with exception of a couple of markers but only in terms of relative ratio): These results prove that the compounds identified are real markers of “freshness” or “not freshness” in egg products and are not related only to a misleading overfitting of the chemometric model.

### 3.5 Markers interpretation

Most of the markers reported in this work have not been previously identified in eggs shelf life studies, because of the analytical approach. While egg freshness is commonly followed by volatile profiling or by e‐nose fingerprinting, the use of high‐resolution liquid chromatography–mass spectrometry allowed to enlarge the identification to medium‐polar nonvolatile compounds.

Volatile markers are actually based on the formation of volatile aldehydes from fatty acids oxidative degradation.^5^ In our study, markers deriving from the very first stage of fatty acid oxidative degradation are reported in the fresh products (namely, 4‐hydroxybutyric acid, hydroxycaproic acid, 2‐hydroxyvaleric acid). These are probably formed during thermal stabilization of egg products, accumulated over time, and further degraded to volatile aldehydes along storage.

Interestingly, most of the compounds reported as markers of freshness are precursors of compounds listed as “markers of nonfreshness.” In particular, it can be observed a strong activation of nitrogen and pyrimidine metabolism pathways, probably on account of the metabolic activity of residual microorganisms growing over time. This leads to the decrease of free amino acids, with the accumulation of biogenic amine (ie, tyramine), amino acid, and purine degradation products (ie, phosphorylethanolamine, *N*‐acetylhistidine, phenyllactic acid, and uracil), and precursors of peptidoglycane (d‐alanyl‐d‐alanine). Consistently, an activation of microbial fermentation is also attested by the accumulation of succinic and lactic acid over time.

## 4 CONCLUSIONS

In the current study, new compounds related to freshness in egg products samples were identified using a nontargeted metabolomic approach. After model creation, the robustness of the identified markers of freshness was assessed through the analysis of a validation set of not previously used egg products.

Along with lactic acid and succinic acid, the reference compounds already considered in the EU legislation, other 29 compounds were detected as discriminant features, allowing a more robust evaluation of freshness.

Further improvements of the results presented in this study should lead to two directions. Firstly, target methods on the identified compounds could be developed for a quantitative evaluation: Quality control laboratories should only be able to detect these 31 molecules, avoiding the need of high‐resolution mass spectrometry and chemometric software, that are much more expensive than a simple single‐stage liquid chromatography–mass spectrometry instrument.

Secondly, this group of compounds, and generally the recorded fingerprints, could be helpful also for both the safety evaluations on microbial growth perspectives and the detection of other frauds related to egg products food chain, as, for example, the illegal use of incubated eggs.
